# Coherent phonon optics in a chip with an electrically controlled active device

**DOI:** 10.1038/srep08279

**Published:** 2015-02-05

**Authors:** Caroline L. Poyser, Andrey V. Akimov, Richard P. Campion, Anthony J. Kent

**Affiliations:** 1 School of Physics and Astronomy, University of Nottingham, Nottingham NG7 2RD, United Kingdom

## Abstract

Phonon optics concerns operations with high-frequency acoustic waves in solid media in a similar way to how traditional optics operates with the light beams (i.e. photons). Phonon optics experiments with coherent terahertz and sub-terahertz phonons promise a revolution in various technical applications related to high-frequency acoustics, imaging, and heat transport. Previously, phonon optics used passive methods for manipulations with propagating phonon beams that did not enable their external control. Here we fabricate a phononic chip, which includes a generator of coherent monochromatic phonons with frequency 378 GHz, a sensitive coherent phonon detector, and an active layer: a doped semiconductor superlattice, with electrical contacts, inserted into the phonon propagation path. In the experiments, we demonstrate the modulation of the coherent phonon flux by an external electrical bias applied to the active layer. Phonon optics using external control broadens the spectrum of prospective applications of phononics on the nanometer scale.

Extensive experimental studies of terahertz (THz) and sub-THz coherent phonons have extended ultrasonic techniques to ever higher frequencies and become the basis for a change-of-paradigm methodology in various areas including high-frequency electronics^1^,^2^, information technology^3^,^4^ biosciences^5^,^6^, and medical diagnostics^7^. Sub-THz phonons are the quanta of acoustic waves having nanometer wavelength, and thus it is possible to reduce the size of acoustic (i.e. phononic) devices, improve spatial resolution of acoustic imaging techniques and increase the speed of acousto-electronic and acousto-optical devices by several orders of magnitude relative to traditional ultrasonics operating at MHz frequencies. Probing nano-objects with coherent sub-THz phonons is an emerging technique, but has already shown its prospective in specific applications such as: hypersonic probing and imaging of nanostructures^8^,^9^,^10^,^11^,^12^,^13^ including biological cells^5^,^6^,^14^,^15^; control of optical^16^,^17^, magnetic^18^,^19^ and microwave-frequency electron transport^1^,^2^,^20^ devices. The typical high-frequency phononic circuits include nanometer phonon generators and detectors fabricated on the same chip, where coherent phonons propagate on macroscopic distances^21^. The ability to manipulate phonon beams and explore “*phonon optics*”, as previously has been achieved for photons, could lead to a revolution in development of new acoustic imaging techniques and methods to control electronic, optical, microwave, quantum and thermal devices which exploit the interaction of phonons with charges, spins and photons^22^.

In the two decades leading up to the 1980s, there was an intensive activity in performing phonon optic experiments^23^ using incoherent acoustic phonons, e.g. heat pulses^24^ and quasi monochromatic phonons generated and detected using superconducting tunnel junctions^25^. These works have contributed significantly to the understanding of fundamental properties of THz phonons, including: anharmonic decay and phonon-phonon interactions; phonon scattering on impurities and defects; and interaction of phonons with other excitations (electrons, photons, magnons etc.)^26^. Phonon optics experiments with coherent sub-THz and THz phonons started later^27^ and showed immediately the prospective for practical applications by extending ultrasonic imaging techniques towards high frequencies and high spatial resolution^28^,^29^,^30^,^31^,^32^. However, methods to control the coherent phonon beams propagating between the phonon generator and detector are limited to “passive” methods (e.g. transmission or reflection of phonons at the single^33^ or multiple interfaces^34^). The ability to control the intensity and the phase of the propagating coherent phonon beams by external means, e.g. by applying an electric field, would be a significant step towards practical applications of sub-THz phonon optics. The present work is concentrated on this challenging task.

In the present work we perform phonon optics experiments on a phononic chip which includes superlattices (SLs): a periodic array of nanometer layers with different acoustic impedances. SLs are widely used for the generation and detection of coherent phonons in phonon optics experiments; they may be made with semiconductors^35^, metals^36^ and soft matter^37^. Furthermore, a doped semiconductor SL under electrical bias turns out to be an *active device* showing a phonon lasing (sasing)^38^ effect and amplification of coherent phonons passing through the biased SL^39^ under appropriate conditions. However, most previous experiments with sub-THz active SLs used bolometric detection of phonons^40^,^41^. This technique, although quite sensitive to the intensity of the phonon flux, does not provide information about the phonon spectrum and phase. Recently, however, we have developed a novel, extremely sensitive, detector for coherent phonons based on a *p-i-n* photodiode device^42^. The high sensitivity and THz bandwidth of this detector have enabled the present work, which is to study a phononic chip including an active SL controlled by external electrical bias. We therefore realize a novel phonon optics experimental setup that integrates, on a single semiconductor chip, a coherent phonon source, a detector of coherent phonons and an active device for electrical manipulation of the phonon beam as it makes its way from the source to the detector. We show that applying electrical bias to the doped SL, the active device, changes the amplitude and phase of the transmitted coherent phonons thus demonstrating the possibility of manipulating the phonon beam intensity with external electrical control.

The paper is organized as follows: after describing the phononic chip used in the experiment, we shall present the results of phonon generation and detection with an unbiased device, which is equivalent to a passive phononic chip. Further, we shall turn to the main experimental result of the work where we study the effect of electrical bias in the doped SL on the amplitude of the transmitted coherent phonon signal. Finally, we present analysis of the results, discussion and conclusions. The experiment and analysis details may be found in the Methods section.

## Results

**Figure 1:**
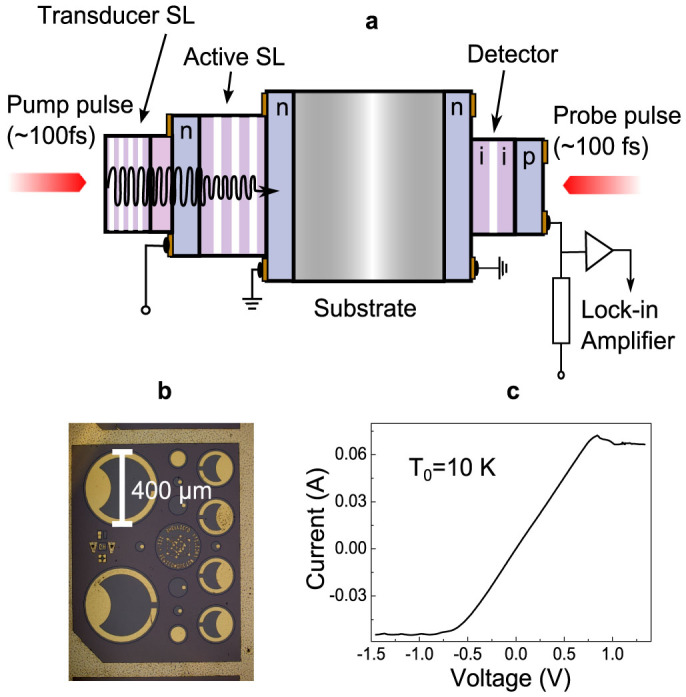
Phononic chip: (a) Coherent monochromatic phonons with *f*_0_ = 387 GHz are generated in the transducer SL when it is excited by optical pump pulses from the femtosecond laser. Phonons pass through the active SL where a bias *V* may be applied, propagate through the 164 μm thick GaAs substrate and are detected in the *p-i-n* diode with femtosecond resolution by means of optical probing from the same laser. (b) A photograph of the phononic chip from the side of the *p-i-n* photodiode. (c) The current-voltage (*I*–*V*) characteristic of the active SL measured at *T*_0_ = 10 K.

The design of the phononic chip and the scheme of the experiment used in the present work is shown in Fig. 1a. The chip consists of three main elements: (i) an undoped GaAs/AlAs transducer SL, in this SL coherent monochromatic phonons with a frequency *f*_0_ = 378 GHz are excited by optical pulses from a femtosecond pulsed laser; (ii) an active n-doped GaAs/AlAs SL with heavily n-doped contacts for applying electrical bias; and (iii) a *p-i-n* detector of coherent phonons on the opposite side of the GaAs substrate to the SLs^42^. The SLs and *p-i-n* on both sides on the substrate were fabricated in the form of mesas (Fig. 1b) with the diameters 400 and 200 μm respectively, centred exactly opposite to each other. The contact pattern on the devices was designed to allow optical accesses for generation of coherent phonons in the transducer SL and probing the phonon beam in *p-i-n* detector. The low temperature current-voltage (*I–V*) characteristic of the active SL device is shown in Fig. 1c. It is linear up to an applied bias of about 500 mV in the negative direction and 800 mV in the positive direction, whereupon oscillations appear due to the formation of space charge domains in the SL^43^. More details concerning the sample fabrication are presented in the Methods section.

In experiments with the phononic chip the generated phonons pass through the active SL, where the bias *V* is applied, then through the GaAs substrate with thickness of *d* = 164 μm, and finally are detected using optical probing with temporal resolution ~1 ps in the *p-i-n* detector. The detected signal *P_V_* (*t*) is monitored as function of the delay *t* between pump and probe pulses. The *P_V_* (*t*) provides direct information about the temporal evolution of dynamical strain in the *p-i-n* which accompanies the coherent acoustic wavepacket. The techniques for generation and detection of coherent phonons are well developed so won't be discussed in detail here, details of the current experiment may be found in the Methods section and earlier papers describing generation in a SL^44^ and detection by a *p-i-n* photodiode^42^. The experiments were performed at the temperature *T*_0_ = 10 K to minimise attenuation of the 380 GHz phonons as they propagate through the substrate. The low temperature is also a requirement for the operation of the active SL, and ensures that the thermal broadening of the electronic energy states is no more than the energy of the 378 GHz phonons. In our experiment the wavelength of pump and probe pulses was chosen in the range 690–700 nm to have sufficient absorption by electron-hole (exciton) optical transition in 4 nm GaAs layers and full transparency in 11 nm AlAs barriers which form the transducer SL. Then theoretical estimations predict the generation of quasi-monochromatic acoustic phonons in the transducer SL with the frequency 380 GHz and the duration of the wavepacket ~4 ns^44^.

### Active SL unbiased

**Figure 2:**
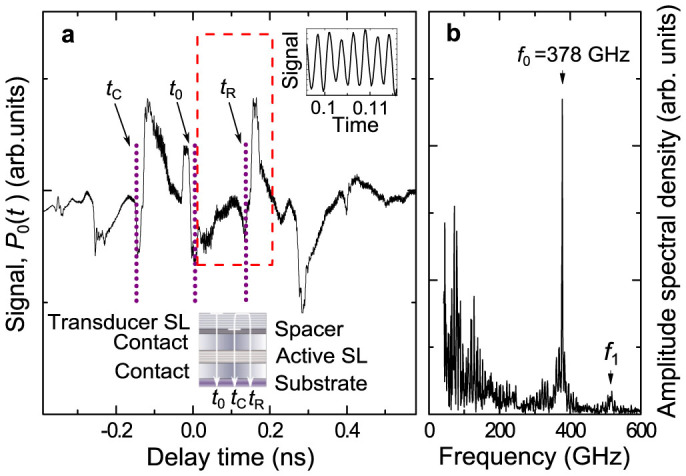
Phonon optics with passive phononic chip. (a) Temporal evolution of the detected signal which accompanies the coherent phonon wavepacket in the *p-i-n* detector for *V* = 0. Sharp peaks, marked by dotted lines, correspond to the strain pulses generated at various interfaces in the phononic chip (the lower inset shows the paths corresponding to the three high amplitude peaks labeled *t*_0_ *t_c_* and *t_R_*). The signal possesses harmonic oscillations with *f*_0_ = 378 GHz (see the zoomed fragment on the upper inset). The dashed rectangle indicates the time interval where harmonic oscillations have maximum amplitude. (b) Fast Fourier Transform of the signal shown in (a) obtained in full time window from −0.3 ns up to 0.5 ns. The sharp peaks and *f*_0_ and *f*_1_ correspond to the coherent monochromatic phonons generated by optical pump pulses in the transducer and active SLs respectively.

With no bias applied to the active SL, the experimental device can be considered to be a passive phononic chip. The temporal evolution of the strain *P*_0_(*t*) detected in the *p-i-n* for the case when the bias is not applied to the active SL (*V* = 0) is shown in Fig. 2a. The temporal trace *P*_0_(*t*) is spread over the time interval ~1 ns and includes several high-amplitude peaks and harmonic oscillations with a frequency of *f*_0_ = 378 GHz (see the zoomed fragment in Fig. 2a). The value *t* = 0 is defined as the arrival time of the phonons generated at the open surface of the top SL to the *p-i-n* detector after their ballistic propagation through the semiconductor chip with the average velocity 4.8 km/s (this path is shown by an arrow t_0_ in the lower inset of Fig. 2a). The fast Fourier transform (FFT) of *P*_0_(*t*) obtained for the full temporal interval is shown in Fig. 2b. The amplitude FFT spectrum consists of a large number of closely located spikes and two well isolated peaks at *f*_0_ = 378 GHz and *f*_1_ = 519 GHz with the widths 2 and 10 GHz respectively indicated in Fig. 2b by the vertical arrows. The temporal shape of *P*_0_(*t*) and its spectrum are in agreement with the earlier results in phonon optics experiments with passive SL devices^21^,^45^. The most important feature is the existence of the essential contribution from monochromatic phonons with the frequency *f*_0_ = 378 GHz that is attributed to coherent phonon generation in the transducer SL as a result of pump optical excitation.

A small fraction of the optical pump beam is transmitted through the contact GaAs layers and reaches the second SL where phonons with *f*_1_ = 519 GHz are generated in the same way as in the top SL. High-amplitude peaks, which form the low frequency part (*f* < 250 GHz) of the phonon spectrum are due to optically induced stress in various semiconductor layers. Particularly, the peaks in *P*_0_(*t*) with the highest amplitudes correspond to the strain pulses generated in the heavily doped n-contact near the interface with the transducer SL. The arrival times *t*_C_ and *t*_R_ of the direct and reflected strain pulses respectively are shown by dotted vertical lines in Fig. 2a and the inset shows the corresponding propagation paths. More details may be found in the Methods section. The spiky character of the whole spectrum is the result of the phonon interference due to multiple reflections at the interfaces and surfaces of the phononic chip.

Further we shall concentrate on the monochromatic part of the phonon spectrum at *f*_0_ = 378 GHz. The pump light penetrates into the top SL and generates coherent phonons throughout its full thickness, and so the first phonons to reach the detector will be emitted from near the bottom of the SL. As the SL is a total of 750 nm in thickness, these phonons arrive 130 ps earlier than those from the top surface. Therefore, the oscillations with frequency *f*_0_ in *P*_0_(*t*) start at ≈-130 ps. These oscillations last for a time longer than 1 ns and possess the highest amplitude in the time interval *t* = 100 – 200 ps marked by dashed rectangle. This interval will be the subject for further studies of the effect of electrical bias in the active SL on the phonon signal at *f*_0_ = 378 GHz.

### Active SL under bias

**Figure 3:**
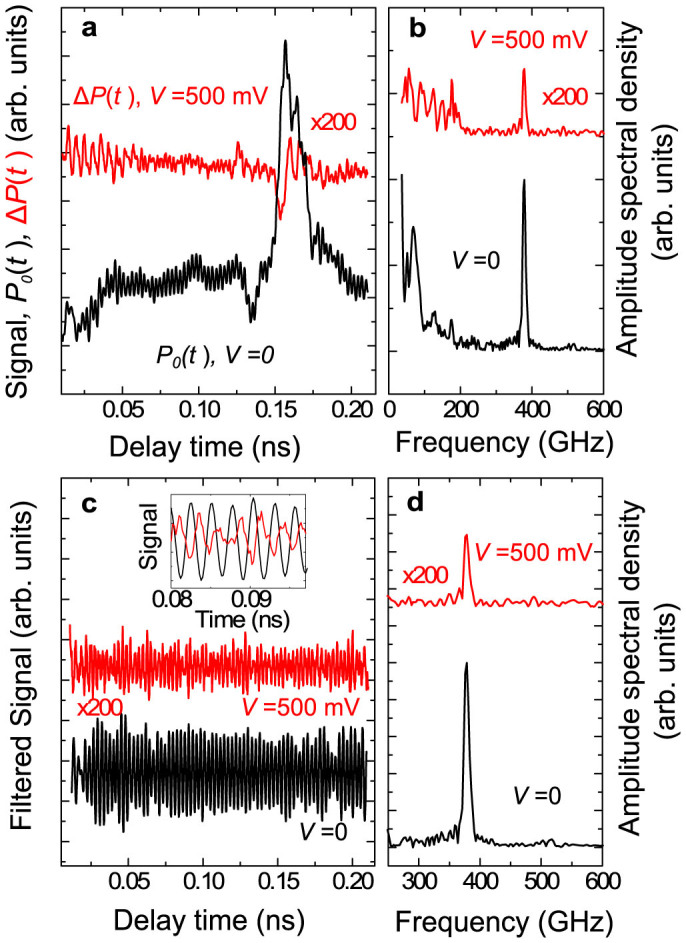
Phonon optics with active phononic chip. (a) Temporal evolution of the bias modulated (modulation amplitude *V* = 500 mV) signal, Δ*P*(*t*) (red line) and the signal *P*_0_(*t*) (black line) measured in passive chip (*V* = 0). (b) Fast Fourier Transforms of the signals shown in (a). (c) and (d) The same as (a) and (b) respectively after applying a digital high pass filtering with the cutoff 250 GHz. The inset in (c) shows the zoomed fragments of the filtered signals, so that the phase difference ΔΨ for harmonic oscillations at *f*_0_ = 378 GHz between Δ*P*(*t*) (red line) and *P*_0_(*t*) (black line) is clearly seen.

To study the effect of applying an electrical bias, *V*, to the doped SL on the coherent phonon signal *P_V_*(*t*) we modulate bias voltage *V* and detect the difference Δ*P*(*t*) = *P_V_*(*t*)-*P*_0_(*t*) using a digital lock-in amplifier. The frequency of the bias modulation was set at 15 kHz, which corresponds to the period much shorter than the thermal time constant of the sample, thus avoiding a significant lattice temperature modulation in the chip. This was confirmed by measuring the arrival time of the acoustic wavepacket with bias on and off as is further explained in the discussion section. We present the results in the time interval marked by dashed frame in Fig. 2a where bias induced effects are the strongest. The measured signals Δ*P*(*t*) for *V* = 500 mV and *P*_0_(*t*) are shown in Fig. 3a. It is seen that both signals, Δ*P*(*t*) and *P*_0_(*t*), possess temporal evolutions which show oscillatory behaviour. The maximum relative changes Δ*P*(*t*)/ *P*_0_(*t*) ~0.14% in the spectral amplitudes at *f*_0_ are high enough to detect the signal in the phononic chip using the *p-i-n* photodiode with sufficient signal to noise level. It is important that the temporal shape of Δ*P*(*t*) is not exactly the same as the temporal shape of *P*_0_(*t*), which means that the origin of Δ*P*(*t*) is not simply due to a modulation of the *p-i-n* detector sensitivity by any stray electric field from the biased SL.

The FFT spectra of *P*_0_(*t*) and Δ*P*(*t*) are shown in Fig. 3b. The two spectra possess some similar features: both consist of the broad low frequency part and the narrow spectral line at *f*_0_ = 378 GHz. The similarity between these two spectra means that the applied bias has an effect on both parts of the spectra: broadband low-frequency part (*f* ≤ 200 GHz) and monochromatic coherent phonons with *f*_0_ = 378 GHz. As mentioned earlier, the major contribution to the broadband low frequency part of the coherent phonon spectrum comes from the strain pulses generated in the *n*-GaAs contact where bias is applied. It is reasonable to assume that the application of bias changes the widths of the depletion regions in the contacts, which affects the optical absorption length and correspondingly the spectrum of the generated coherent phonons in the acoustic wavepacket of the strain pulse. This effect, while being interesting by itself, however stays beyond the present work, and further we shall concentrate on the contribution from monochromatic phonons with *f*_0_ = 378 GHz to Δ*P*(*t*). The only source of these monochromatic phonons in the studied phononic chip is the transducer SL excited by the optical pump pulses.

For clarity of the presentation we apply high-pass digital filtering to *P*_0_(*t*) and Δ*P*(*t*) transmitted phonon spectra components with cut off frequency at 250 GHz, and show corresponding traces and spectra in Figs. 3c and 3d respectively. It is seen that after filtering the harmonic oscillations with *f*_0_ = 378 GHz look more distinct and it is possible to compare the phases of the monochromatic contributions in *P*_0_(*t*) and Δ*P*(*t*) (see inset in Fig. 3c). Fitting the temporal curves with a harmonic function ~sin(2*πf*_0_*t* + Ψ) it is possible to obtain the phase difference ΔΨ = Ψ_0_ − Ψ_Δ_ where Ψ_0_ and Ψ_Δ_ are the phases of the *f*_0_ harmonic component *P*_0_(*t*) and Δ*P*(*t*) respectively. Performing the fitting procedure in seven 20 ps temporal intervals we get a value of ΔΨ = (0.76 ± 0.03)π which will be discussed in more detail in the next section.

## Discussion

We start the discussion by considering the relative amplitude and phase of *f*_0 _components in the signals *P*_0_(*t*) and Δ*P*(*t*). Ignoring any nonlinear effects it is easy to show that the amplitude ratio α = *D*/*A*_0_ (*A*_0_ and *D* are the amplitudes of the *P*_0_(*t*) and Δ*P*(*t*) respectively) depends on the bias induced changes of Δ*A* = *A_V_-A*_0_ (*A_V_* is the amplitude of *P_V_*(*t*)) and on the difference Δ*t* between the arrival times of coherent phonons to the *p-i-n* detector with bias on and off. From the experiment we estimate *α* ~ 0.14% for *V* = ± 500 mV and, using the analysis described in the methods section, we deduce that |Δ*A*/*A*_0_| ~ 0.1% and Δ*t* ~ 0.4 fs. These results show the extremely high sensitivity of the modulation technique and *p-i-n* phonon detection used in this experiment.

The non-zero value of Δ*t* is most likely to be due to the bias induced modulation of the sound velocity as a result of lattice temperature modulation. Indeed, application of bias to the active SL is accompanied by Joule heat and respectively by an increase of the lattice temperature in the propagation path for phonons from the transducer SL to the *p-i-n* detector. From the difference in the arrival time of the phonon induced signal at *V* = 0 and 0.5 V applied bias, we estimate the average sample temperature *T*_1_ based on the dependence of the sound velocity *c* on *T* in GaAs, i.e. 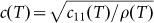, where *c*_11_ and *ρ* are the elastic constant and density of GaAs respectively. Using the published temperature dependencies of *c*_11_^46^ and *ρ*^47^, we estimate that the average sample temperature is raised from *T*_0_ = 10 K to the value *T*_1_ ≈ 23 K by applying the bias.

Modulation of bias with a frequency of 15 kHz leads to modulation of the sample temperature with the amplitude Δ*T*, which, for the case when the modulation period is much less than the thermal time constant of the sample, is (≪ *T*_1_). Assuming the measured value of the delay Δ*t* = 0.4 fs is purely due to the temperature modulation, then using the value 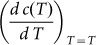, we can estimate Δ*T* ~ 10^−3^ K. Such a small value of Δ*T* cannot explain the measured value |Δ*A*/*A*_0_| if the transmission change was due to anharmonic phonon-phonon scattering. Indeed, estimates based on Herring mechanism of anharmonic decay^48^ give relative changes of the *f*_0_ phonon amplitude of not more than 4 × 10^−5^ which is two orders of magnitude smaller than the experimentally measured |Δ*A*/*A*_0_| ~ 1 × 10^−3^. Thus we exclude the modulation of the lattice temperature as the reason for the bias induced modulation of the phonon flux amplitude.

**Figure 4:**
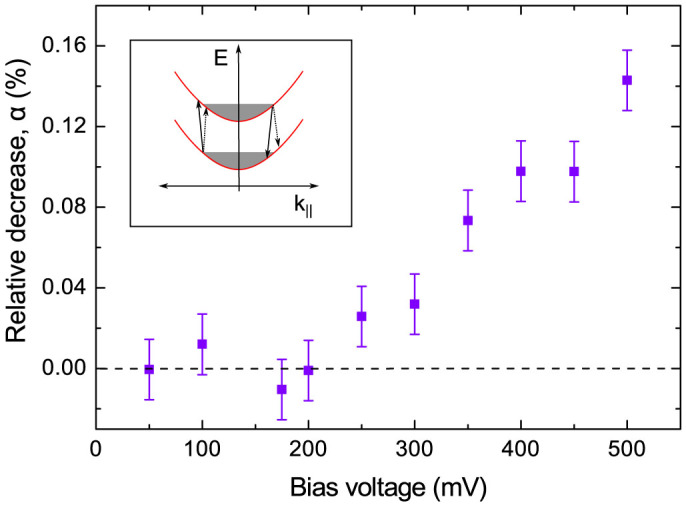
Electron-Phonon interaction in the active SL. The dependence of the amplitude of the spectral peak at *f*_0_ = 378 GHz in bias modulated signal Δ*P*(*t*) on the applied bias *V*. The inset shows schematically the phonon assisted transitions for electron tunneling between the neighboring GaAs layers in the Stark ladder. Solid and dotted black arrows indicate transitions with absorption or stimulated emission of phonons respectively.

Our explanation of the bias-induced modulation of the coherent phonon flux with *f*_0_ = 378 GHz is based on the model of electron-phonon interactions in the active SL. The band diagram of the SL with applied bias, where electrons are well separated in energy and space, is called a Stark ladder^49^. The electrons are mostly confined within the GaAs layers, but are able to tunnel via the AlAs barriers giving rise to electrical conduction in the direction perpendicular to the SL layers. Electron transitions between states can occur with the absorption or emission of phonons within a GaAs layer, or between neighbouring GaAs layers, respectively known as intra- and inter-quantum well (QW) transitions. The coherent phonons generated and detected in the chip propagate almost exactly along the direction normal to the active SL layers. In this direction intra-QW transitions are effectively cut off for phonons of frequency 378 GHz due to the momentum conservation condition^50^. The two possible phonon-induced inter-QW transitions involve stimulated absorption and stimulated emission, as illustrated in the inset of Fig. 4^51^. Although the active SL structure is similar to those for which evidence of sasing has previously been observed^40^,^41^, the higher level of doping used, and hence higher electron density, is such that population inversion throughout the active SL is not achieved under the conditions of the experiment. Therefore the stimulated absorption of phonons is expected to be the dominant process for 378 GHz phonons, and the flux will decrease as we measure experimentally.

The dependence of the relative decrease of *α* on *V*, shown on Fig. 4, is an increasing function. It is important that the dependence of *α* on *V* is very different from the *I–V* characteristic of the active SL (see Fig. 1c) thus serving as an additional argument for excluding experimental artefacts, like modulation of sensitivity of the detector by *V*.

A possible qualitative explanation for the measured dependence of *α* on *V* is that, with increasing bias, the electron temperature, *T*_e_, in the active SL increases. The effect of this is to increase the number of possible near-vertical electron transitions involving electron states within a region ~ *kT*_e_ of the Fermi energy. This might explain the reasonable fit to the data of a function proportional to the bias dependence of the electrical power dissipated in the active SL. However, for a complete description of the dependence of *α* on *V*, a more detailed analysis in respect to the specific model of inhomogeneity and high doping in the studied SL is required, which goes well beyond the scope of the present paper.

Finally, we have performed a phonon optics experiment with the possibility to control the flux of the coherent sub-THz phonons by applying external electric field to the doped semiconductor SL inserted in the phononic chip. The layer with the doped SL serves as an active element in the coherent phonon propagation path from the generator to detector. The relative amplitude change of the demonstrated coherent phonon flux modulation has a value ~10^−3^, which, although small, was detected with reliable signal to noise level by sensitive detector for coherent phonons based on the *p-i-n* photodiode. With further work to optimise the structure and doping of the active SL, we believe it should be possible to increase the depth of modulation. Phononic chips with coherent detectors and generators separated by a macroscopic distance which can be controlled externally have a potential for exploiting THz and sub-THz acoustics in acousto-electrical, acousto-optical, and thermal devices. It is already believed that coherent phonon optics based on passive devices may cause “Sound and Heat Revolutions in Phononics”^22^. Inserting active elements in the phononic chips controlled externally will enable much broader possibilities for phonon control on the nanometer scale and related prospective applications.

## Methods

### Phononic chip

The semiconductor layers in the chip shown in Fig. 1(a) were grown by molecular beam epitaxy (MBE) on both sides of a 164 μm-thick (001) semi insulating GaAs substrate. The active SL consists of 50 periods, each period comprised of 5.9 nm GaAs and 3.9 nm of AlAs, n-doped with Si to a concentration of 10^17^ cm^−3^. At each end of this are 0.5 μm-thick, electrical contact layers n-doped with Si to a concentration of 10^18^ cm^−3^. The contact layers are separated from the active SL by 20.2 nm thick GaAs layers in which the doping is linearly graded from 10^17^ to 10^18^ cm^−3^. On top of the active SL structure, and separated from it by a 100 nm undoped GaAs spacer, is a transducer SL consisting of 50 periods, each of 4 nm GaAs and 11 nm of AlAs. The transducer SL emits a flux of coherent phonons when excited by a femtosecond laser pulse with a photon energy greater than the lowest energy of electron-hole resonance in the GaAs layers, 1.76eV in this case. The calculated frequency of the dominant phonon mode in the phonon flux is given by *f_0_* ≈ *c*/*d_SL_* = 380 GHz (*c* is the speed of longitudinal sound in the SL and *d*_SL_ is the SL period), which is well away from the phononic stop bands of the active SL. The phonon detector based on the *p-i-n* photodiode was grown by MBE on the opposite side of the substrate. The phonon detector consists of 3 period SL with 1.2 nm of GaAs and 1 nm of Al_0.33_Ga_0.77_ As. It is grown in the middle of a 200 nm-thick undoped (*i*-intrinsic) Al_0.33_Ga_0.77_ As layer and has maximum sensitivity to phonons when probed with the optical pulses with photon energy ~1.76 eV, close to the band gap of GaAs layers in the SL. The *i*-region is embedded between 200 nm thick contacts from n-doped (10^18^ cm^−3^ Si) Al_0.33_Ga_0.77_ As and p-doped (10^18^ cm^−3^ C) Al_0.33_Ga_0.77_ As layers. This particular structure was chosen so that its optical resonance matched the optical resonance of the phonon transducer SL, and it was found to be at least as sensitive as the single GaAs quantum well based structure^42^.

### Pumping and probing the phonon signal

Optical pump and probe beams came from a tuneable mode locked Ti:sapphire ~100 fs pulsed laser with a repetition frequency of 82 MHz operating at a wavelength of 700 nm. After passing through the motorized optical delay line the pump beam was focused on the transducer SL to the 50 μm diameter spot and the maximum energy density at the surface of the phononic chip was ~50 μJ/cm^2^ per pulse. The probe beam was focused on the *p-i-n* detector to the spot with diameter ~20 μm. The amplified photocurrent of the biased *p-i-n* was detected by the lock-in amplifier referenced to 15 kHz from the acousto-optical modulator in the path of the pump beam (in experiments with *V* = 0) or from the pulses controlling the bias modulation in the active SL.

### Generation of strain pulses at the interfaces in the phononic chip

The signal *P*_0_(*t*) shows a number of sharp peaks. Three of them are explained in the caption of Fig. 2 and the related text. The temporal shape *P_0_(t)* also possesses other peaks with smaller amplitudes which may be explained by the arrival of the strain pulses generated at various interfaces due to stress induced by optical pump excitation. The total number of peaks also includes the echoes in the *p-i-n* diode due to the phonon reflection at the open surface from the side of phonon detector^42^. All these peaks form the lower frequency part of the phonon spectrum detected by the *p-i-n* photodiode and shown in Fig. 2 (b) and do not influence the conclusions of the present work.

### Analysis

We checked experimentally measuring *P_V_*(*t*) applying dc bias *V* (without bias modulation) that Δ*A/A* and 2*πf*_0_Δ*t* are both much less than unity. We may get the following equation for the ratio α:
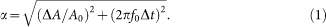
When Δ*t* = 0 (no bias induced changes in the arrival time), then *α* = |Δ*A/A*_0_| and the phase shift ΔΨ = 0 or ΔΨ = *π* when the applied bias induces the increase or the decrease of the amplitude of harmonic oscillations respectively. In the other extreme case, when Δ*A* = 0, α = |2*πf*_0_Δ*t*| and ΔΨ = ± *π/*2. In more the general case, Δ*t*≠0 and Δ*A*≠0, the phase difference is equal to:
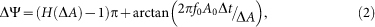
where *H*(*x*) is the Heaviside function. Eq.(2) allows one to conclude that the experimentally measured value ΔΨ = 0.76π may be realized only if Δ*A* < 0 and this means a decrease of the oscillation amplitude when bias is applied to the active SL. Using the experimentally measured value of ΔΨ and Eq.(2) we may estimate Δ*t*. Following this Eq.(1) and the experimentally measured value of α may be used to estimate (ΔA/A_0_).

## Author Contributions

A.J.K. and A.V.A. developed the idea for the experiment, C.L.P. and A.V.A. performed the experiment, C.L.P., A.V.A. and A.J.K. analysed the experimental data and wrote the manuscript, R.P.C. fabricated the samples.
